# Meiotic double-strand DNA breaks and spontaneous mutation in *Drosophila melanogaster*

**DOI:** 10.1093/g3journal/jkag019

**Published:** 2026-01-23

**Authors:** Rob Melde, Austin Daigle, JoHanna Abraham, Nathaniel Sharp

**Affiliations:** Department of Genetics, University of Wisconsin—Madison, Madison, WI 53706, United States; Department of Genetics, University of North Carolina at Chapel Hill, Chapel Hill, NC 27599, United States; Department of Genetics, University of Wisconsin—Madison, Madison, WI 53706, United States; Department of Genetics, University of Wisconsin—Madison, Madison, WI 53706, United States

## Abstract

The exchange of genetic material during meiosis requires the formation and repair of DNA double-strand breaks (DSBs), which may not be repaired with perfect fidelity. If meiotic exchange is mutagenic, this would add to the costs of sexual reproduction and affect patterns of genome evolution, but much of the evidence for this is indirect. In the fruit fly *Drosophila melanogaster*, it is possible to completely suppress endogenous DSBs while retaining normal fertility. We took advantage of this system to generate fly strains with and without a mutant allele of *mei-P22*, a gene that is essential for meiotic DSB formation, on a common genetic background. This allowed us to investigate the relationship between DSBs and genome-wide mutation patterns, using a mutation accumulation design to allow unselected spontaneous mutations to be observed. Following 30 generations of mutation accumulation, we identified over 1,800 mutations by whole-genome sequencing. The presence of meiotic DSBs had little effect on the rate and spectrum of point mutations. We found that mutations were more likely to occur in areas of the genome with higher rates of crossover recombination, regardless of whether meiotic DSBs were occurring. We also found that the rate of transposable element insertions across multiple TE families was substantially elevated in the group lacking meiotic DSBs, suggesting that host suppression of mobile genetic elements is closely associated with meiotic recombination mechanisms.

## Introduction

Meiotic recombination (MR) is a key driving force of evolution. MR is ubiquitous across eukaryotes and is a core aspect of sexual reproduction. While MR is generally considered evolutionarily favorable (Keightley and Otto 2006), these benefits come with costly tradeoffs. One obvious cost of MR is the time and energy cells must spend to perform this process. For example, in the model yeast *Saccharomyces cerevisiae*, the mitotic cell cycle takes approximately 90 min under optimal conditions, whereas meiosis and sporulation require at least 8–12 h to complete (Ray and Ye, 2013; Duina et al. 2014 ). From a population genetics perspective, beneficial alleles that work well in concert with one another can be broken apart by MR, slowing adaptation (Charlesworth and Charlesworth 1975; Charlesworth and Barton 1996). At the molecular level, while MR typically leads to proper chromosome segregation, errors in this process can lead to chromosomal nondisjunction and aneuploidy (Koehler et al. 1996). Another potential molecular cost relates to the formation and repair of meiotic double-strand DNA breaks (DSBs), which are required for MR. Some DSBs occur spontaneously as a result of DNA damage; these events, described as “non-programmed” or “exogenous” DSBs, are considered highly mutagenic because error-prone pathways are commonly used to repair them (Strathern et al. 1995; Rodgers and McVey 2016). In contrast, the “endogenous” or “programmed” DSBs that occur during meiosis are thought be more tightly regulated and repaired using specialized pathways (Longhese et al. 2009; Wei et al. 2019). Nevertheless, endogenous DSBs likely contribute at least some additional risk of mutation, and there is some evidence for such an effect.

A positive correlation between crossover recombination rate and molecular diversity may be an indirect source of evidence on the relationship between endogenous DSBs and mutation, but this pattern is not observed in all species, and can instead be explained by reduced background selection in areas of higher crossover recombination (Begun and Aquadro 1992; Smukowski and Noor 2011; Cutter and Payseur 2013 ). Stronger evidence of a relationship between MR and mutation comes from humans, where SNPs and *de novo* germline mutations are found more commonly near known recombination hotspots (Pratto et al. 2014; Hinch et al. 2023). Similarly, short insertions and deletions (INDELs) and structural variants have been shown to be associated with recombination hotspots in human pedigree studies (Beyter et al. 2021; Hinch et al. 2023). Parent-offspring sequencing has also revealed an association between crossover events and germline mutations in humans (Halldorsson et al. 2019) and to a lesser extent in bees (Yang et al. 2015; Liu et al. 2017). Parent-offspring sequence data in *Drosophila melanogaster* and *D. simulans* did not detect any relationship between crossovers and germline mutations, but the number of relevant phased mutations was low (14 female-derived mutations) (Wang et al. 2023). In *S. cerevisiae*, meiosis is associated with a higher mutation rate than mitosis (Magni 1963), and this effect has been directly linked to endogenous DSBs (Rattray et al. 2015). Additional direct tests of the impact of MR on the rate and spectrum of point mutations would be valuable.

Another mutational process associated with recombination is the insertion of transposable element (TE) sequences. TEs are found more often in genomic regions of low recombination (Petrov et al. 2011; Kofler et al. 2012; Kent et al. 2017). The Hill-Robertson effect and selection against ectopic recombination could drive this pattern (Biemont et al. 1997; Dolgin and Charlesworth 2008; Kelleher et al. 2020), but there is also evidence that TEs suppress recombination (Huang et al. 2025). The formation of endogenous DSBs has been shown to be related to both TE mobility and TE silencing (Dufourt et al. 2014; Wylie et al. 2016; Wei et al. 2019). This suggests that altering the formation of endogenous DSBs could influence TE mobility in an activating or suppressing manner, but this possibility has not been thoroughly explored.

Quantifying the mutational consequences of endogenous DSBs is challenging. In many organisms, endogenous DSBs are essential for proper chromosomal segregation and gamete formation (Petronczki et al. 2003). In such systems, researchers are typically limited to comparing mutations between mitotically and meiotically dividing cells or examining mutational patterns near crossover events and recombination hotspots, which are approaches with significant limitations. Comparing mitotic vs meiotic cells confounds the effects of DSBs with the broader differences between fundamentally distinct cell division processes. Additionally, focusing on crossover-associated mutations provides only a partial view of mutations that may be associated with endogenous DSBs, since many meiotic DSBs are resolved without crossing over. These methodological constraints therefore limit our understanding of how endogenous DSBs truly impact the overall mutational landscape.

Conveniently, *D. melanogaster* can perform meiosis and form viable gametes without endogenous DSBs. In nearly all *Drosophila* species, males naturally perform meiosis without endogenous DSBs or crossover recombination (Lee and Rosin 2024). Laboratory strains have been developed where meiotic DSBs are also absent in the female germline. One example contains the allele *mei-P22[P22]* (also known as *mei-P22[1], mei-P22[McKim]* and henceforth referred to as *mei-P22^−^*), generated with P-element mutagenesis (Sekelsky et al. 1999). The *mei-P22* protein forms a dimer with *mei-W68* (a paralog of *spo-11*) and interacts with *Trem*, *Vilya,* and *Narya* to form endogenous DSBs (Liu et al. 2002; Mehrotra and McKim 2006; Lake et al. 2011, 2015, 2019). The *mei-P22^−^* allele effectively eliminates transcription of *mei-P22*, preventing endogenous DSB formation and MR in the female germline while permitting viable gamete production (McKim et al. 1998; Liu et al. 2002; Iida and Lilly 2004). Importantly, the effect of *mei-P22^−^* is distinct from that of balancer chromosomes, which inhibit crossing over but do not prevent DSBs (Miller et al. 2016).

In *D. melanogaster* females, meiosis involves about six crossovers throughout the genome, but a total of 20–24 endogenous DSBs that must be repaired (Carpenter 1987; Mehrotra and McKim 2006). Compared to organisms with recombination hotspots, the landscape of crossing over in *D. melanogaster* females is relatively uniform. The correlation between mutation and recombination found in humans may be more apparent than such a relationship in flies would be, because there are specific 1–2 kb regions where recombination (and thus endogenous DSBs) tend to occur, mediated by PRDM9 hotspots (Jeffreys et al. 2001; Baudat et al. 2010). Endogenous DSBs in *D. melanogaster* have only been observed to occur within euchromatin, and are influenced by accessible chromatin states linked to active transcription but are otherwise broadly distributed throughout the genome (Mehrotra and McKim 2006; Adrian and Comeron 2013; Adrian et al. 2016 ).

To study the effect of meiotic DSBs on mutation, we established two sets of mutation accumulation (MA) lines: one using females undergoing normal endogenous DSB formation, and another using *mei-P22*^−^ females in which endogenous DSBs were absent. In MA experiments, repeated bottlenecking minimizes the efficacy of natural selection, allowing most new mutations to become fixed with neutral probability (reviewed in Katju and Bergthorsson 2019). Such experiments are a powerful means of generating large, unbiased mutation datasets with relatively modest sequencing effort. We used this approach to compare the pattern of germline mutation in the presence and absence of endogenous meiotic DSBs. We found that eliminating endogenous DSBs had little impact on the rate and spectrum of point mutations but caused a significant increase in the rate of TE insertion throughout the genome.

## Methods

### Fly stocks and mutation accumulation

We reared flies on defined medium (14.3 g/L agar, 92.3 g/L white sugar, 46 g/L debittered yeast, 7.4 g/L potassium sodium tartrate, 0.93 g/L potassium phosphate, 0.46 g/L sodium chloride, 0.46 g/L calcium chloride, 0.46 g/L magnesium chloride, 0.46 g/L iron sulfate, 0.5% propionic acid) in standard vials seeded with live yeast, incubating at 25 C with a 12-hour light:dark cycle. We obtained stock 4931 from the Bloomington Drosophila Stock Center (RRID:BDSC_4931) which is *y*^1^  *w*^1^/Dp(1;Y)*y*^+^; P{*w*[ + mC] = lacW}*mei-P22^P22^*; *sv^spa-pol^*. To ensure a fair comparison of the mutation patterns between treatment groups, we sought to establish MA lines with and without *mei-P22^−^* from the same genetic background. To create these lines we first backcrossed *mei-P22^−^* into a stock of *w*^1^  *y*^1^/Dp(1;Y)*y*^+^ for five generations; *mei-P22^−^* can be tracked in these crosses because it is marked with a mini-white allele, which partially reverses the eye color effect of *w*^1^ (*mei-P22^−^* does not eliminate crossing over in the heterozygous state). In this genetic background, XX females are *y*^1^/*y*^1^ and express a yellow body color phenotype; XY males are *y*^1^/Dp(1;Y)*y*^+^, where Dp(1;Y)*y*^+^ marks the Y chromosome with a functional copy of *y*, such that males express a wild-type body color. The presence of *mei-P22^−^* increases the rate of non-disjunction (Sekelsky et al. 1999; Liu et al. 2002), resulting in viable XXY females and sterile XØ males, which can be identified by body color (wild-type body color in XXY females and yellow body color in XØ males). After backcrossing for five generations, we imposed single-pair bottlenecks for an additional five generations to remove much of the standing genetic variation. We then performed a final set of expansion crosses, selecting either for or against *mei-P22*^−^, establishing 20 lines in each treatment group. For MA, each line was bottlenecked for 30 consecutive generations using one randomly selected male and one randomly selected female, excluding XXY and XØ flies. Five lines went extinct during the course of the experiment, which ultimately concluded with 16 lines where endogenous DSBs occurred (*mei-P22*^+^) and 19 lines where they did not (*mei-P22*^−^).

### DNA extraction and sequencing

Upon completion of the 30th generation of MA, we collected all female offspring (6–24 individuals per line) and froze them at −80 C. We extracted genomic DNA from stored flies using the Qiagen DNeasy Blood & Tissue Kit with the insect protocol (catalog #69504), purified using the Qiagen DNeasy PowerClean Kit (catalog #12877-50), and quantified using a Qubit Fluorometer (ThermoFisher Scientific). We submitted DNA to the University of Wisconsin Biotechnology Center DNA Sequencing Core Facility (RRID:SCR_017759) for paired-end whole genome sequencing using an Illumina NovaSeq X Plus.

### Calling point mutations

We obtained about 2.3 billion reads in total, which we quality checked using FastQC (v0.12.1) and trimmed using Trimmomatic (v0.39) (Andrews 2010; Bolger et al. 2014). After masking repetitive regions using RepeatMasker (v4.1.5), we mapped reads to the *D. melanogaster* reference genome (v6.59) using bwa-mem2 (v2.2.1) (Smit et al. 2013; Dos Santos et al. 2015; Vasimuddin et al. 2019), resulting in a mean depth of coverage of 99. We sorted and indexed BAM files using Samtools (v1.6), removed duplicate reads using Picard Tools MarkDuplicates (v3.1.1), and called variants using GATK HaplotypeCaller (v4.5.0.0) (Li et al. 2009; Broad Institute 2019). Finally, we used RStudio (v4.3.2) for variant filtering, statistical modeling, and figure creation (Van der Auwera and O’Connor 2020; R Core Team 2023). We utilized glmmTMB (v1.1.11; Brooks et al. 2017) to fit generalized linear mixed-effect models of genome-wide mutation patterns.

To filter our callset, we first selected variants that were only present in one line and required that the site of interest have a called genotype in all lines (finding the exact same mutation in two or more lines due to convergent mutation is highly unlikely, and such cases should represent sequencing errors or preexisting variation). We required there to be at least 10 supporting reads for the variant allele and considered only variants passing the filters recommended by GATK: mapping quality ≥ 50, quality by depth ≥ 2, Fisher strand bias ≤ 60, strand odds ratio ≤ 3, absolute mapping quality rank sum ≤ 8, and absolute read position rank sum ≤ 4. We additionally required that the depth at a variant site was no less than half and no greater than twice the median chromosome-wide depth in that line. Finally, we removed any single-nucleotide variants (SNV) or INDELs called within 1 kb of a structural variant (SV) or TE insertion. Where possible, we applied the same filtering criteria to non-variant sites when calculating the number of callable sites in the genome. For a site to be considered callable, we required that site to pass the filters on all lines. We considered variants only on the major contigs listed in the *D. melanogaster* reference genome that are present in female flies, and that normally recombine (2L, 2R, 3L, 3R, and X; variants called on chromosome 4 are also listed in Supplementary Material). The reference genome for these contigs includes 133,880,608 sites, and our final callset includes 99,458,130 sites (74%). Of the 34,422,478 non-callable sites, 20,052,895 were not considered callable because they were deemed repetitive by RepeatMasker. The remaining 14,369,583 sites were considered non-callable because they failed one or more of our filtering metrics. We categorized events as single nucleotide substitutions (SNV), short insertions and deletions (INDEL), multinucleotide variants (MNV; two or more SNV within 1 kb in the same line), and complex variants (COMPLEX; two or more variants within 1 kb in the same line where at least one variant was an INDEL). For the purposes of mutation rate estimation, each MNV and COMPLEX group of variants was counted only once. To understand the predicted functional impacts of the variants in our dataset, we used Ensemble's variant effect predictor (release 113) to identify the most severe effect predicted for each variant (McLaren et al. 2016). We set the upstream/downstream distance to zero and kept all other parameters at their defaults.

We estimated point mutation rates using both homozygous and heterozygous variants, and using an adjusted number of MA generations (Supplementary Material), since more than one copy of a given chromosome is effectively included in each line (Supplementary Fig. 5). We did not find evidence that the proportion of mutations that are heterozygous differs between treatment groups (Results); the adjustment of generation number affects absolute mutation rates but not treatment differences. Performing our statistical comparisons using only homozygous mutations did not alter our conclusions (Supplementary R script).

### Calling structural variants

To identify larger structural variants that may have been missed by HaplotypeCaller, we used MANTA (v.1.6.0) (X. Chen et al. 2016). For practical purposes, we analyzed our 35 samples in 3 blocks (10, 10, and 15 samples) and then combined the outputs. Each analysis block had lines from both treatment groups. We then filtered variants by selecting only those that were present in one line with a called genotype for every line present in that block. We removed any variants that did not have at least 10 supporting spanning reads or 10 supporting split reads, and considered only cases with the maximum possible quality score.

### Calling transposable element variants

We used the McClintock meta-pipeline (v.2.0.0) to detect TE insertions and excisions using multiple detection methods (J. Nelson et al. 2017; Chen et al. 2023), specifically, the McClintock implementation of TEMP2 (Yu et al. 2021 ) employing the default settings from McClintock. Because TE callers rely on information from discordant read pairs, where one read in the pair maps to the reference genome and the other maps to a TE sequence, several studies have shown that trimming reads can improve the detection accuracy of these tools, despite a reduction in coverage depth (Yu et al. 2021; Daigle et al. 2025). This is especially relevant for our dataset, where the median insert size (about 300 bp) is close to the total length of read pairs (150 × 2 bp), which will lead to many overlapping pairs. For this reason, we trimmed all reads at the 3′ end down to 50 bp using fastp (J. Chen et al. 2023) before TE detection. We used the consensus TE sequences and reference genome annotation created by Rech *et al*. (available at http://doi.org/10.20350/DIGITALCSIC/13765 and http://doi.org/10.20350/DIGITALCSIC/13894, respectively) as inputs to McClintock (Rech et al. 2022).

To identify non-reference TE insertions unique to each MA line, we compared the sets of predicted insertions across all samples. Because TE callers often report slightly different breakpoint coordinates for the same insertion (Chen et al. 2023; Daigle et al. 2025), we treated insertions in different lines from the same TE family located within 50 bp of one another as representing the same event. Using this criterion, we identified TE insertions confidently present in only one MA line, which we interpreted as *de novo* mutations specific to that lineage. We excluded non-reference insertions present in more than one line, as it is unclear if these are the result of detection error, recurrent mutation TE insertions at the same site, or variants that remained segregating in the stocks used to create the MA lines. TEMP2 additionally checks for the presence or absence of reference TE sequences. We interpreted reference TEs uniquely absent in one MA line as de novo TE excision events. However, we note that we do not have the ability to distinguish between a deletion that removes a TE and a true enzymatic excision of a TE.

### Analysis of genome-wide mutation patterns

We used statistical models to test whether specific genomic features could account for variation in the locations of mutations throughout the genome, and whether these patterns differed between the two treatment groups. We considered crossover recombination rates, replication timing, chromatin state information, and G/C content. We used 7,657 genomic windows defined by chromatin state (Filion et al. 2010), and added information on crossover recombination rates (Comeron et al. 2012) and replication timing (Schwaiger et al. 2009) for each window, using weighted averaging to account for the different window sizes for each variable. We used the Kc cell type replication timing data in our analyses (Schwaiger et al. 2009), but note that the Kc and Cl8 cell type replication timing values are strongly correlated (*P* < 2.2 × 10^–16^; *R^2^* = 0.812). For each window, we used a custom bash script to count the number of sites that were considered callable in our dataset as well as the total number of G/C sites in that window according to the reference genome. The chromatin state data includes five categories (Filion et al. 2010), with two being distinct types of euchromatin, referred to as “red” (RE) and “yellow” (YE), two being distinct types of heterochromatin, referred to as “blue” (BL) and “green” (GR), and one being a highly repressed type of chromatin, referred to as “black” (BK). The GR heterochromatin is defined by the presence of heterochromatin protein 1 and the methylation of lysine 9 of histone H3, whereas the BL heterochromatin is associated with polycomb group proteins and methylation of lysine 27 of histone H3. BK chromatin is highly repressed, makes up about half of the *D. melanogaster* genome, and is defined not only by virtually no gene expression, but by the proteins histone H1, D1, IAL, and SUUR. Finally, RE and YE chromatin share characteristics often associated with regions that are actively transcribed (high levels of H3K4me2, H3K79me3, and low levels of H3K9me2 and H3K27me3). They differ in that RE chromatin is uniquely marked in a variety of ways, including ATPase Brahma, SU(VAR)2-10, MED31, CAF1, several histone-modifying complexes and DNA-binding factors, GAGA factor, and Jun-related antigen. YE chromatin is predominantly marked by MRG15 and H3K36me3 (for additional details on each chromatin type, see Filion et al. 2010). The replication timing and chromatin state datasets were built under an older release of the *D. melanogaster* reference genome, so we used the FlyBase Drosophila Sequence Coordinates Converter (https://flybase.org/convert/coordinates) to convert coordinates to reference version r6. We excluded chromosomes Y and 4, any windows missing replication timing or recombination data, and any windows with zero callable sites from our analysis.

This windowed approach allowed us to run generalized mixed-effect models with the number of variants observed in that window as the response variable and genomic features as predictors. We scaled the recombination rate and G/C content to their respective means to facilitate model convergence. We also included power (the number of callable sites multiplied by the number of lines, scaled linearly) as either an additional predictor or an offset term. To compare treatment groups, we duplicated the window data, included treatment as a predictor, and included window identity as a random effect. We found that including the chromosome as a random effect did not improve model fit. We used negative binomial generalized linear mixed effect models (GLMMs) with the nbinom2 family (Hardin and Hilbe 2007) implemented using glmmTMB (Brooks et al. 2017), which accounts for any overdispersion.

## Results

### Point mutation rate

**Fig. 1.**
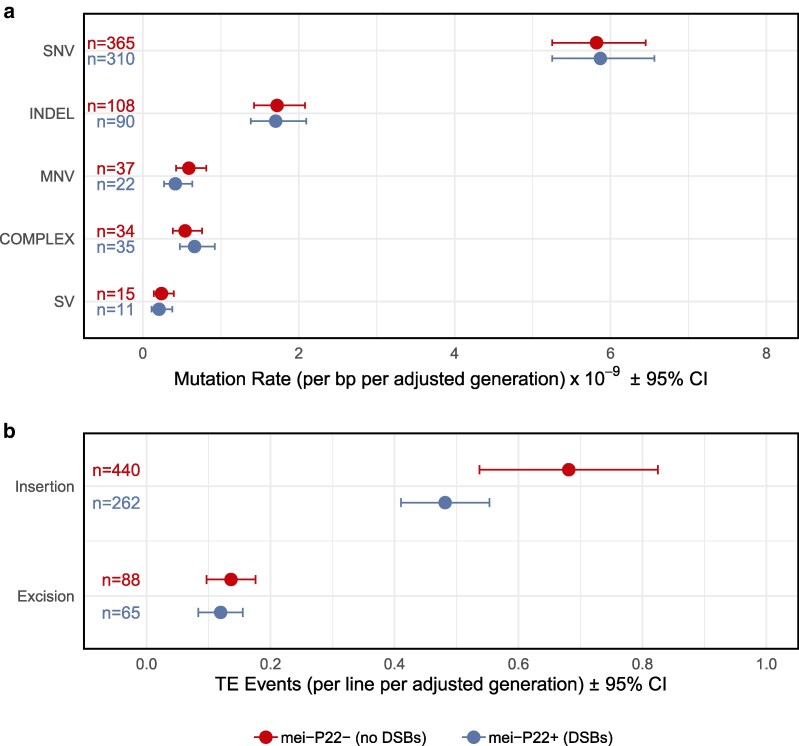
Rates of mutation by group and mutation type. Error bars represent 95% confidence intervals. a) Rates of single-nucleotide variants (SNVs), short insertions and deletions (INDEL), multinucleotide variants (MNVs), complex variants (COMPLEX), and structural variants (SVs) per callable site per generation. b) Transposable element (TE) insertions and excisions per line per generation.

We identified a total of 991 point mutations across 35 MA lines that accumulated mutations for 30 generations (Fig. 1a). The presence of meiotic DSBs did not detectably influence the rate of any point mutation type (SNV: t-test, *P* = 0.92; INDEL: t-test, *P* = 0.95; MNV: Wilcoxon rank sum test, *P* = 0.25; COMPLEX: Wilcoxon rank sum test, *P* = 0.24; Table 1), or the distribution of point mutations among chromosome arms (χ^2^ = 1.36, *P* = 0.85). The overall point mutation rate did not vary significantly among chromosome arms (χ^2^ = 4.11, *P* = 0.39), or on the X chromosome vs autosomes (binomial test, *P* = 0.45). The majority of the point mutations we identified were in the homozygous state (55.3%), as expected given that MA took place under full-sib mating. We found that the frequency of homozygous vs heterozygous variants did not differ significantly between groups (Fisher's exact test, *P* = 0.11; Supplementary Fig. 1). Regardless of the treatment group, variants on the X chromosome were more likely to be homozygous than those on the autosomes (Fisher's exact test, *P* = 2.57 × 10^–4^). We found no evidence that the amount of variance among MA lines differed between treatment groups for SNVs, INDELs, MNVs, COMPLEXs, or SVs (Levene tests, all *P* > 0.25).

**Table 1.** Rates for each mutation type across experimental groups and groupwise differences.

Type	Mutation Rate Estimate		95% Confidence Interval		Absolute Group Difference (%)
	Endogenous DSBs	No Endogenous DSBs	Endogenous DSBs	No Endogenous DSBs	
SNV	5.87 × 10^−9^	5.82 × 10^−9^	(5.25 × 10^−9–^6.56 × 10^−9^)	(5.25 × 10^−9–^6.45 × 10^−9^)	0.86
INDEL	1.70 × 10^−9^	1.72 × 10^−9^	(1.38 × 10^−9–^2.10 × 10^−9^)	(1.43 × 10^−9–^2.08 × 10^−9^)	1.17
MNV	4.17 × 10^−10^	5.90 × 10^−10^	(2.71 × 10^−10–^6.34 × 10^−10^)	(4.26 × 10^−10–^8.16 × 10^−10^)	34.36
COMPLEX	6.63 × 10^−10^	5.42 × 10^−10^	(4.74 × 10^−10–^9.25 × 10^−10^)	(3.85 × 10^−10–^7.60 × 10^−10^)	20.08
SV	2.08 × 10^−10^	2.39 × 10^−10^	(1.11 × 10^−10–^3.78 × 10^−10^)	(1.41 × 10^−10–^3.98 × 10^−10^)	13.87
TE Insertion	4.82 × 10^−1^	6.81 × 10^−1^	4.10 × 10^−1–^5.53 × 10^−1^	5.37 × 10^−1–^8.25 × 10^−1^	34.22*
TE Excision	1.19 × 10^−1^	1.36 × 10^−1^	8.36 × 10^−2–^1.55 × 10^−1^	9.67 × 10^−2–^1.76 × 10^−1^	13.33

An asterisk indicates a difference that is statistically significant. The only statistically significant difference is the rate of TE insertion. Note that for SNV, INDEL, MNV, COMPLEX, and SV rates, values are reported in units of per base pair per generation, whereas for TE insertions and excisions, values are reported in units of per genome per generation.

### Transposable element activity

**Fig. 2.**
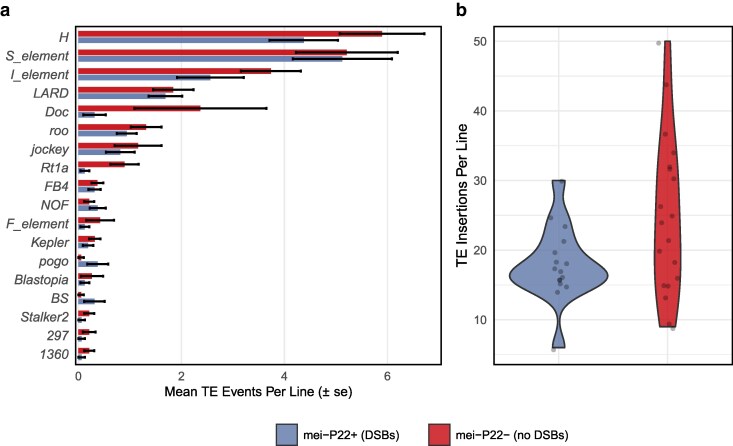
Transposable element activity by family and treatment group. a) Only TE families with at least five novel insertions or excisions across both treatment groups and at least one insertion/excision in each group are shown here. A complete list is given in Supplementary Material. b) Points represent each MA line where the number of TE insertions is plotted. Points are separated by group.

We detected 855 cases of TE movement, of which 82% were insertions. We found a significant difference between treatments in the average number of TE events per line (Welch's t-test, *t* = 2.35, *P* = 0.027), where the group with endogenous DSBs averaged 0.68 events per line per generation and the group without endogenous DSBs averaged 0.93 TE events per line per generation (Fig. 1b). This result persists when putative TE excisions are excluded (Welch t-test, *t* = 2.43, *P* = 0.022), but not when considering excisions alone (Welch t-test, t = 0.61, *P* = 0.543). We found significantly more variance in the number of TE insertions per MA line in the group that did not perform endogenous DSBs (Levene's Test, *P* = 7.15 × 10^–3^), but little evidence for a difference in terms of coefficient of variation (bootstrap *P* = 0.083). The group without endogenous DSBs therefore incurred more TE insertion events per generation, with increased among-line variance attributable to the increased mean. In both treatment groups, we observed a significantly higher proportion of TE insertions on the X chromosome than expected based on chromosome size (binomial tests; with endogenous DSBs: *P* = 1.41 × 10^–7^; without endogenous DSBs: *P* = 1.07 × 10^–9^). We detected TEs from numerous different “families” (Fig. 2a), including S elements (long inverted repeat DNA transposon), H elements (hAT superfamily DNA transposon), Doc elements (non-LTR LINE retrotransposon), and roo elements (LTR retrotransposon). The increased rate of transposition we observed in the lines lacking endogenous DSBs was evident in most of these TE families (Fig. 2a). We did not observe exceptional TE insertion rates in any particular MA line (Fig. 2b). To test whether the insertion rates of different TE families were associated with one another, we determined the number of families showing any activity in each MA line, regardless of the insertion count; the distribution of family counts did not differ significantly from the Poisson expectation (χ^2^ = 14.6, simulated *P* = 0.60). Similarly, we found that the insertion counts for the five most active TE families were not correlated with one another across MA lines (ten Spearman rank correlations: range: −0.27 to 0.29, all *P* > 0.096). These results suggest that TE families were mobilizing independently of one another.

### Structural variants

Based on sequencing coverage, we identified one *mei-P22^−^* MA line with apparent trisomy of chromosome 4 (Supplementary Fig. 2). We also identified 11 large deletions, 13 large insertions, and 2 tandem duplications for a total of 26 structural variants (size 265–7799 bp). The presence of meiotic DSBs did not influence the rate or average length of SVs (Wilcoxon rank sum test, *P* = 0.34; Welch *t*-test, *t* = 0.11, *P* = 0.91). The distribution of structural variants across chromosome arms did not differ from the null expectation (χ^2^ = 6.52, *P* = 0.16).

### Mutation spectrum

**Fig. 3.**
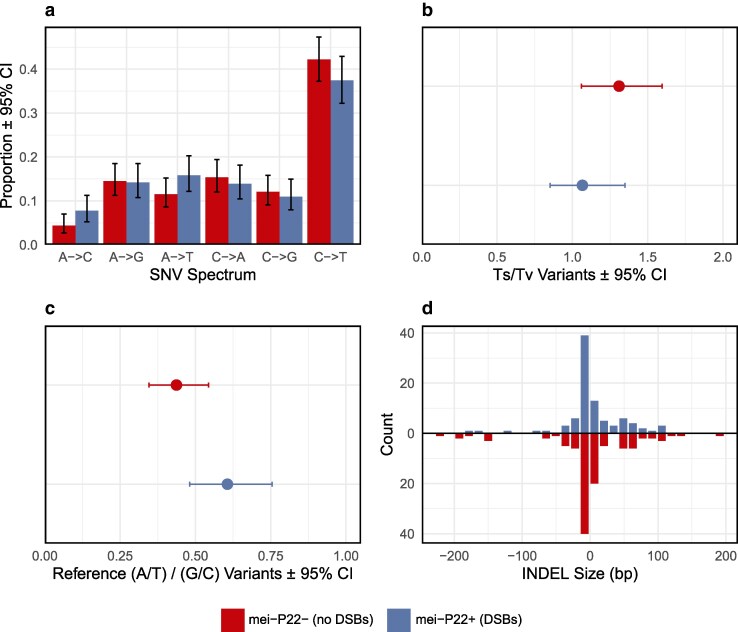
Point mutation spectrum in each treatment group. a) The spectrum of single-nucleotide variants. 95% confidence intervals reflect Wilson score intervals for multinomial proportions. b) The ratio of transitions to transversions with bootstrap 95% confidence intervals. c) The ratio of variants that occurred at A/T sites vs G/C sites, with bootstrap 95% confidence intervals. d) Length distribution of short insertions and deletions.

The presence of meiotic DSBs did not significantly influence the SNV spectrum (Fig. 3a; χ^2^ = 6.87, *P* = 0.23) or the transition-transversion ratio (Fig. 3b, Fisher's exact test, *P* = 0.19). In both treatment groups, SNVs were much more likely to occur at G/C sites than at A/T sites (binomial tests, all *P* < 3.85 × 10^–11^). However, we found some evidence that the strength of this effect differed between treatments (χ^2^ = 4.03, *P* = 0.045), with greater bias toward mutation at G/C sites in the group without meiotic DSBs (69.59% of SNVs vs 62.26%, Fig. 3c). The INDEL mutations in our dataset showed a deletion bias (Fig. 3d; binomial test, *P* = 5.84 × 10^–4^), with no difference in this bias between treatment groups (Fisher's exact test, *P* = 0.53). The median deletion size was 6 bp, and the median insertion size was 15 bp, unaffected by treatment (Wilcoxon rank sum tests, all *P* > 0.30).

**Fig. 4.**
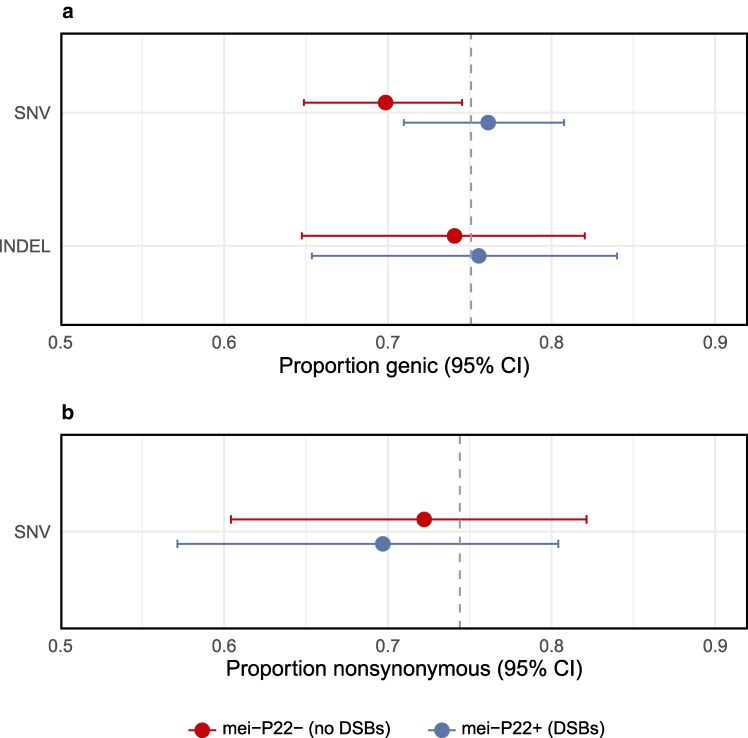
Genomic consequences of point mutations. a) Proportion of variants found within genes (including introns). The gray dashed line is the proportion of callable sites in our dataset that were genic. b) Proportion of coding SNVs that were nonsynonymous. The gray dashed line is the null value calculated in Sharp et al. (2016).

To ensure that selection did not bias our results, we examined the frequency of mutations in coding regions and their consequences for protein sequences. We found that 72.7% of SNVs and 74.4% of INDELs occurred within genes (including introns; Fig. 4a), which does not significantly differ from a null expectation of 75.08% for our callable sites (binomial tests, *P* = 0.17 and *P* = 0.94, respectively). Similarly, 71.0% of genic SNVs were nonsynonymous, which does not differ significantly from the expected value of 74.4% (binomial test, *P* = 0.38; Fig. 4b). Additionally, these metrics did not differ between treatment groups (Fisher's exact tests of genic frequency; SNV: *P* = 0.07; INDEL: *P* = 0.87; Fisher's exact test of nonsynonymous SNVs: *P* = 0.85). We therefore find no evidence that selection biased the accumulation of mutations overall or affected the treatment groups differently.

### Genomic context of mutations

**Fig. 5.**
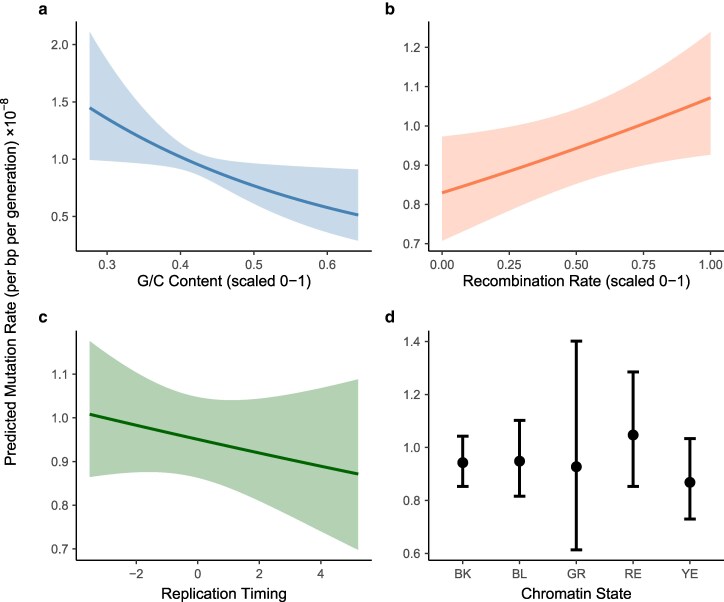
Relationships between point mutations and genomic context. Results are based on regression analysis of 7,657 genomic windows, combining data across treatment groups, with 95% error bands. Point mutations were more likely to occur in regions of lower G/C content a) and in regions of high crossover recombination b). Point mutations were not significantly associated with replication timing c) or chromatin state d).

**Fig. 6.**
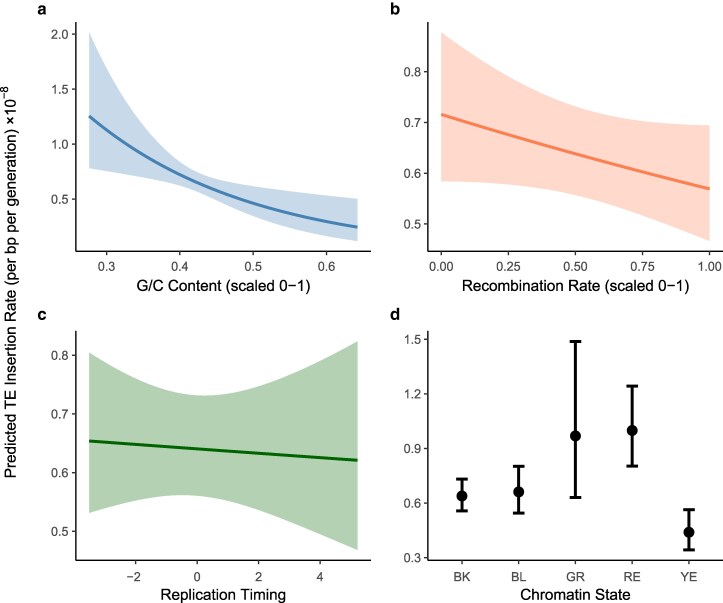
Relationships between transposable element activity and genomic context. Results are based on regression analysis of 7,657 genomic windows, combining data across treatment groups, with 95% error bands. TE insertions were associated with lower G/C content a) and chromatin state d). TE insertions were not significantly associated with crossover recombination rate b) or replication timing c).

The presence of endogenous DSBs could affect disproportionately mutation patterns in specific regions of the genome. Considering models of point mutations and TE activity across 7,657 genomic windows, we found no evidence that treatment group interacted with G/C content, crossover recombination rate, chromatin state, or replication timing in determining the rate of mutation (likelihood ratio tests, all *P* > 0.15). Combining treatment groups, point mutations were associated with lower G/C content (*z* = −2.19, *P* = 0.028) and higher crossover recombination rates (*z* = 2.19, *P* = 0.028), with no significant effect of replication timing (*z* = −0.89, *P* = 0.37) or chromatin state (likelihood ratio test, *P* = 0.80) (Fig. 5). TE activity was associated with lower G/C content (*z* = −2.76, *P* = 5.75 × 10^–3^) and differed significantly among chromatin states (likelihood ratio test, *P* = 0.04), but did not vary with crossover recombination rate (*z* = −1.52, *P* = 0.13) or replication timing (*z* = −0.25, *P* = 0.80) (Fig. 6).

## Discussion

Eliminating endogenous meiotic DSBs appeared to have little effect on the overall rate of point mutations, but a substantial effect on the rate of transposition (Fig. 1). Our experimental design focused on genome-wide mutation patterns in the presence or absence of meiotic DSBs, rather than on mutations occurring at known or controlled sites of meiotic exchange. This approach permitted detection of “global” effects—transposition throughout the genome—but likely limited our power to detect mutation patterns associated with specific DSB locations. While not significant, we do find that the rate of single-nucleotide changes was greater in flies with endogenous DSBs, as expected if DSB repair is mutagenic. If we assume 24 meiotic DSBs per genome per generation (Carpenter 1987; Mehrotra and McKim 2006), and that each repair tract involves the synthesis of 5000 bp (Adams et al. 2003), our point estimates would be consistent with an increased mutation rate within such tracts of 6.7-fold.

We considered whether the presence of endogenous DSBs might disproportionately affect point mutation patterns in specific regions of the genome, particularly euchromatic regions where DSBs are more likely to occur, or areas of the genome showing higher levels of crossover recombination; we did not detect significant interactions with treatment for either of these factors. Instead, we found that point mutations were associated with regions of higher crossing over regardless of the presence of meiotic DSBs (Fig. 5), suggesting that some regions of the genome are more likely to experience both meiotic exchange and mutation, although the underlying mechanism for this is unclear. Such a pattern would contribute to the association between nucleotide diversity and crossover rate, independently of any mutagenic effect of DSBs. Importantly, we did not attempt to call point mutations in repetitive regions, which likely have distinct mutation patterns (Nishant et al. 2009; Flynn et al. 2018; López-Cortegano et al. 2025). An additional caveat to this analysis is that it incorporates data collected in separate experiments using cell lines and inbred strains that may or may not reflect the genomic features of the flies we studied.

An increased risk of mutation during the resolution of DSBs could be mitigated if the proteins that create DSBs also facilitate DNA repair, including the repair of exogenous, non-programmed DSBs. The suppression of endogenous DSBs in *mei-P22^−^* flies could limit the activity of DSB-dependent repair mechanisms such as the GATOR complex, potentially dampening responses to exogenous DNA damage (Wei et al. 2019 ), and counteracting any anti-mutagenic effect of eliminating endogenous DSBs.

In contrast to point mutations, we found a clear effect of our treatment on TE activity, with the rate of novel TE insertions increasing by 41% in *mei-P22^−^* flies (Fig. 1b). This increase was not restricted to a particular TE family (Fig. 2a), specific MA line (Supplementary Fig. 3) or genomic context; TE events were associated with lower G/C content and certain chromatin states regardless of treatment (Fig. 6). One possible interpretation of this pattern is that *mei-P22^−^* flies were “stressed”, inducing TE activity, but only specific forms of stress appear to have such an effect in *Drosophila* (Sharp and Agrawal 2016; de Oliveira et al. 2021; Mombach et al. 2022; Milyaeva et al. 2023; Merenciano et al. 2025). Another alternative explanation is that preexisting copies of TEs in the region surrounding the backcrossed mei-P22 allele were present in the group with increased TE activity and were responsible for driving the observed effect. Due to the nature of backcrossing an allele into different genetic backgrounds, our two groups do differ with respect to the number of preexisting TE copies on chromosome 3L (the location of mei-P22). This variation includes some of the commonly active TE families observed in our experiment, such as Doc, H, I, F, Rt1a, and roo elements (Supplementary Fig. 4). The number of unique copies found in each of these families in this region ranges from one to seven, with most families having more preexisting copies in the lines that performed endogenous DSBs. While this preexisting genetic variation could affect patterns of MA, the endogenous DSB group—which started with more unique TE copies in this region—displayed significantly less overall TE activity, so we find this alternative explanation unlikely. Instead, our results are consistent with previous observations linking TE activity with meiotic DSBs. Alleles disrupting the GATOR complex, which is involved in DSB repair, cause increased levels of TE expression (Wei et al. 2019), and alleles preventing proper synaptonemal complex formation result in fewer meiotic DSBs and increased TE insertion genome-wide (Miller 2020). There is also a trend toward increased transposition in males, where endogenous DSBs are absent (Wang et al. 2023). TE repression may therefore be connected with the generation and/or repair of meiotic DSBs.

We found that TE insertions were more likely to occur on the X chromosome, in G/C poor regions, and in certain chromatin states (Fig. 6), matching previous findings (Adrion et al. 2017). However, the insertion bias toward certain chromatin states does not seem to be based simply on accessibility; insertion rates in the two types of euchromatin, RE and YE, both differed significantly from the highly repressed BK chromatin, but did so in opposite directions (Fig. 6). The continual improvement of bioinformatic tools for detecting TE movement will aid further investigation into complex insertion site preferences and distinguishing genomic patterns of TE mutation vs selection (Sultana et al. 2017; Zhang et al. 2020).

In conclusion, our results indicate that point mutations arising due to meiotic DSB repair represent a small fraction of the genome-wide mutational burden in *D. melanogaster*, and that spatial correlations between mutation and recombination may occur even in the absence of meiotic DSB repair. Our study also adds to the growing evidence that MR in this organism is tightly associated with the suppression of TE activity.

## Supplementary Material

jkag019_Supplementary_Data

## Data Availability

Whole genome sequence data is available from the NCBI SRA under bioproject accession number PRJNA1346113. The R script used for analyses and its subsequent R workspace, our point mutation callset, TE callset, SV callset, the genomic windows used in analyses, and supplemental figures are available through Dryad DOI: 10.5061/dryad.b2rbnzstx. Supplemental material available at G3 online.
