# Supplementary material for: Meiotic double-strand DNA breaks and spontaneous mutation in Drosophila melanogaster
Source: G3 (Bethesda). 2026 Jan 23;16(4):jkag019. doi: 10.1093/g3journal/jkag019 (PMC13042288; doi:10.1093/g3journal/jkag019)
Supplement: jkag019_Supplementary_Data [file jkag019_supplementary_data.docx]

**Supplementary Information**

When MA is conducted by full-sib mating, as opposed to clonal propagation (as in microbes) or tracking single non-recombining chromosomes (as in some *Drosophila* MA experiments), simply dividing the number of mutations by the number of generations (multiplied by the number of sites and lines) does not result in an accurate mutation rate estimate, especially when MA took place over relatively few generations. If both homozygous and heterozygous variants are included, the mutation rate will be overestimated because more than one copy of each chromosome is effectively being included in the numerator but not the denominator. If only homozygous variants are included, the mutation rate will be underestimated because not all true mutations will become homozygous by the time of sequencing.

To estimate mutation rate with precision, we developed a correction based on the rate of heterozygosity (*H*) decay under full-sib mating. Given heterozygosity in the first two generations of *H*_0_ = 1 and *H*_1_ = 1, expected heterozygosity in subsequent generations is given by *H_t_* = 0.5 *H_t_*_–1_ + 0.25 *H_t_*_–2_ (Crow and Kimura, 1970). Using this recursion equation, the “effective” number of generations of MA is approximately:

$$t^{*}=t+\sum_{1}^{t} H_{t}$$

In the limit, when MA is conducted over many generations, *t** ≈ *t* + 5, including when *t* = 30, as in our experiment. To test this correction, we performed simulations of mutation accumulation using the number of lines and generations of our experiment, using a true mutation rate of 6 × 10^–9^ and 1.32 × 10^8^ sites per haploid genome, allowing for chromosome segregation and optional recombination (one recombination event per chromosome arm per generation), with one random individual “sequenced” from each line at the end of MA. We performed 5000 simulations with and without recombination and estimated mutation rates using either *t* or *t**. As expected, without the correction, mutation rates were overestimated by 16.7%, on average, with no overlap with the true rate, regardless of recombination (Figure S5). Using the corrected generation number, mutation rate estimates were on average indistinguishable from the true rate (bias <0.02%, *P* > 0.98). We therefore proceeded to use *t** to obtain corrected mutation rate estimates.

**References**

Crow JF and Kimura M. *An introduction to population genetics theory.* 1970. Blackburn Press.

**Supplementary Figures**


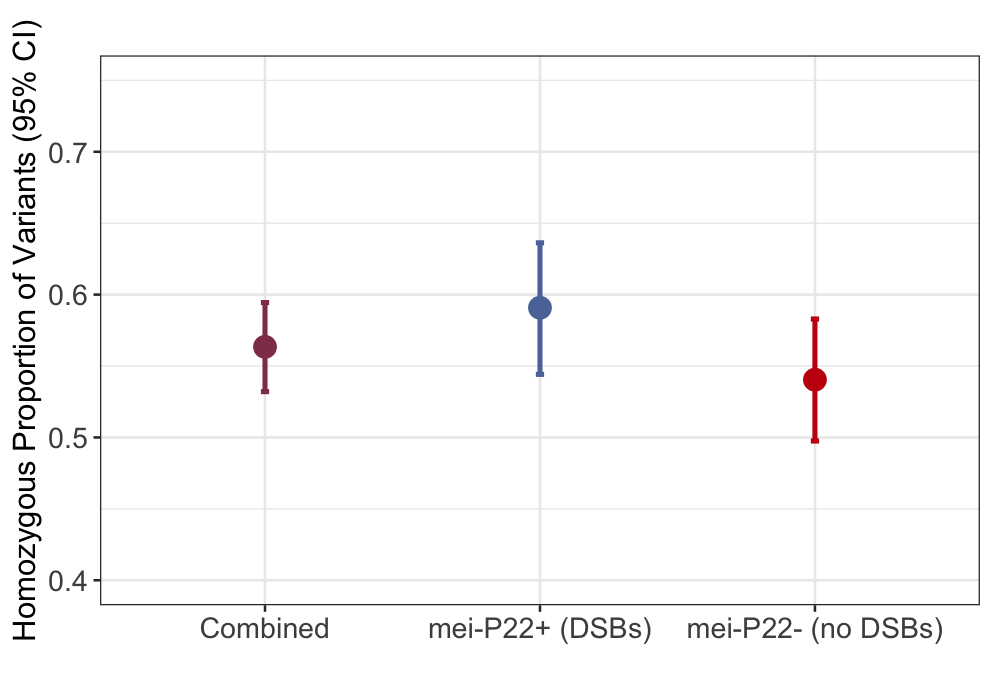


**Figure S1. Proportion of point mutations detected in the homozygous state.** Given the full-sibling mating design of our MA experiment, we expect many new mutations to become homozygous by the time of genome sequencing, following 30 bottleneck generations, but heterozygous mutations will still be present that have not yet become fixed.


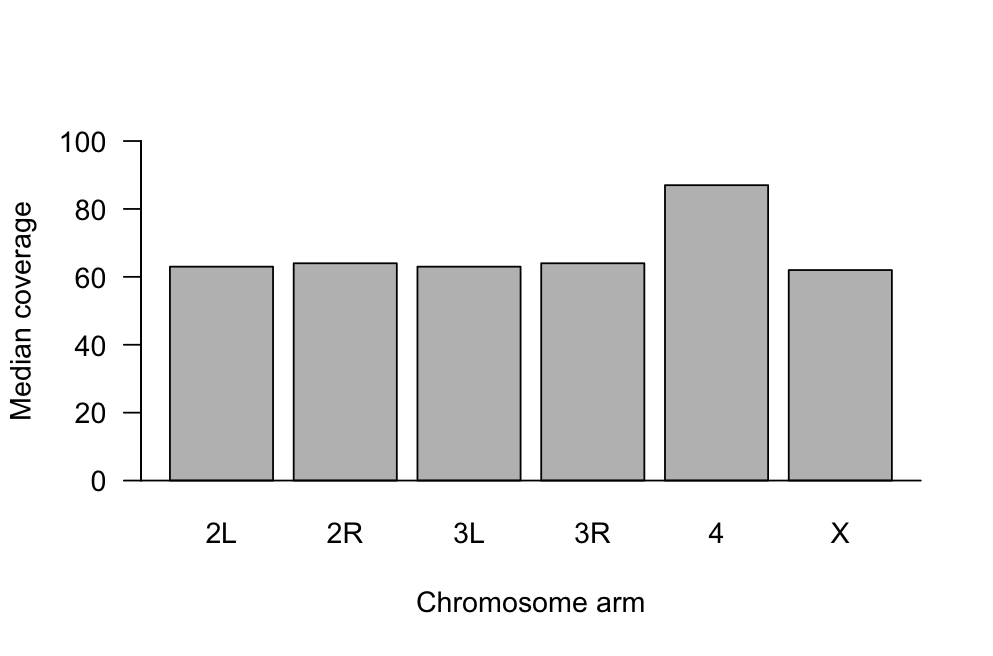
**Figure S2. Median coverage by chromosome arm for MA line 11.** In this line, the elevated median coverage on chromosome 4 (1.38-times the genome-wide median) likely indicates trisomy.

**Figure S3. TE insertions by family across each individual MA line.** The heatmap shows each experimental lines on the y-axis and each TE family in which we observed at least one new insertion on the x-axis.

**Figure S4.** **TE insertion-frequency differences across chromosome arm 3L (0–13 Mb) between MR and no_MR accumulation lines.** Vertical lines show, for each TE insertion locus detected by TEMP2, the difference in estimated population frequency between groups, plotted as Δ = MR_freq − no_MR_freq, where MR_freq and no_MR_freq are the fractions of lines in each group carrying a call overlapping that locus. Positive values (Δ > 0) indicate loci observed more frequently in MR lines; negative values (Δ < 0) indicate loci observed more frequently in no_MR lines; Δ ≈ 0 indicates similar frequencies in both groups. A dashed vertical line marks the position of the mei-P22 allele, which corresponds to a P-element insertion at 3L:7,243,977–7,244,457 (black star). Loci belonging to H, I_element, Doc, roo, Rt1a, and F_element are highlighted with colored triangles when the locus is present in at least 20% of lines in either group (max(MR_freq, no_MR_freq) ≥ 0.2). Upward triangles denote MR-enriched loci (Δ > 0) and downward triangles denote no_MR-enriched loci (Δ < 0). All other loci remain visible as lines.

**Figure S5. Simulations to test a correction factor for the number of generations of MA under full-sib mating.** (A) Simulations with recombination. (B) Simulations without recombination. In both cases, the uncorrected values overestimate the true mutation rate, whereas the corrected values are centered on the true mutation rate.
